# Underground guardians: how collagen and chitin amendments shape soil microbiome structure and function for *Meloidogyne enterolobii* control

**DOI:** 10.1186/s40168-025-02132-8

**Published:** 2025-06-12

**Authors:** Josephine M. Pasche, Roshni Sawlani, Victor Hugo Buttrós, Johan Desaeger, Karen A. Garrett, Samuel J. Martins

**Affiliations:** 1 Department of Plant Pathology, University of Florida, Gainesville, FL 32611 USA; 2 Department of Entomology and Nematology, Gulf Coast Research and Education Center, University of Florida, Wimauma, FL 33598 USA; 3 Global Food Systems Institute, University of Florida, Gainesville, FL 32611 USA

**Keywords:** Rhizosphere, Bacteria, Biocontrol, Disease management, Soil ecology, Root-knot nematodes

## Abstract

**Background:**

The emergence of the guava root-knot nematode (*Meloidogyne enterolobii*) poses a significant threat to tomato yields globally. This study evaluated the impact of collagen and chitin soil amendments on soil microbial composition and function (fungal and bacterial communities) and their effects on tomato plant health and *M. enterolobii* infection under standard (5000 eggs plant^−1^) and high (50,000 eggs plant^−1^) inoculum pressure. Conducted in a greenhouse setting, the study investigated the effectiveness of these amendments in nurturing beneficial microbial communities across both native and agricultural soils.

**Results:**

Both collagen and chitin were effective in reducing nematode egg counts by up to 66% and 84% under standard and high inoculum pressure, respectively, and enhanced plant health parameters (biomass and chlorophyll content). Moreover, a microbiome shift led to an increase in bacterial (*Kitasatospora*, *Bacillus*, and *Streptomyces*) and fungal (*Phialemonium*) genera, known for their chitinase, collagenase, and plant-parasitic nematode control. Among the microbes, *Streptomyces* spp. were found among the core microbiome and associated with a lower disease incidence assessed through a phenotype-OTU network analysis (PhONA). Under standard inoculum, higher metabolite expression was observed with amino acids representing a majority among the metabolite groups.

**Conclusions:**

The findings highlight the potential of collagen and chitin to mitigate *M. enterolobii* infection by fostering beneficial soil microbial communities.

Video Abstract

**Supplementary Information:**

The online version contains supplementary material available at 10.1186/s40168-025-02132-8.

## Background

The root-knot nematode, *Meloidogyne enterolobii*, is a global threat to a broad range of plants, including a wide variety of tomato cultivars, with the damage from this species alone causing up to 65% yield loss [1]. The use of chemical controls such as nematicides may not be an economically viable management option, due to high costs and negative impacts on the environment and human health [2]. These issues lead to the increasing need for alternatives to chemical control. The use of more sustainable management approaches, such as the use of beneficial microbes, has been shown to be effective in promoting plant health and suppressing plant diseases [3]. Beneficial microbes have also been shown to be effective specifically in controlling infection by *Meloidogyne* spp. [4–6].

Microbes can lead to an antagonistic effect against nematodes, and soil amendments may be used to favor beneficial microbes over others [4]. Approximately, 80% of the nematode cuticle is composed of collagen, and with the addition of collagen to the soil, bacteria that produce collagenases are likely to accumulate [7, 8]. The collagenase activity may damage the nematode cuticle, resulting in decreased nematode populations in the roots of tomato plants [9, 10]. Likewise, chitin is an essential component in the eggs of nematodes, and chitinase-producing microorganisms may have an impact on the presence of nematode infections. H. Tian, R. D. Riggs, and D. L. Crippen [11] isolated chitinolytic bacteria by adding chitin (1% w/w) to the soil of soybean plants in a greenhouse trial, allowing the bacteria to inhabit the soil, and then isolating with selective media. These isolates were tested in vivo and effectively controlled nematode (*Heterodera glycines*) infections. In this study, we hypothesized that soil amendment with collagen and chitin will alter soil microbial assemblages to favor beneficial microbes, decrease nematode infection, and contribute to overall plant health.

The objectives of this study are as follows: (1) Assess the amendments (collagen and chitin) effects on tomato plant biomass, chlorophyll content, and severity of guava root-knot nematode infection under two different inoculum pressures, (2) determine the effects of collagen and chitin amendments on soil microbial composition by analyzing fungal and bacterial microbiome networks and identifying which microbes are favored, (3) assess the core microbiome constituents that persist in the rhizosphere across different soil types and inoculum pressures, identify organisms associated with lower disease incidence, and analyze the changes in soil metabolites.

## Methods

### Soil collection

Two soils were used in this experiment: a native soil and an agricultural soil. The agricultural soil was from an organic tomato field, with successive tomato cultivation located at the University of Florida Gulf Coast Research and Education Center, Wimauma, FL, USA, field (39.6-m altitude, 27°45′23″ N, 82°13′29″ W). Between seasons, the cover crop Bahia grass (*Paspalum notatum*) was planted in the agricultural soil, and the fertilization additions were performed during bedding following the fertility recommendations for tomato in the Florida Vegetable Production Handbook [12]. The native soil was collected from an uncultivated area covered in native foliage approximately 9 m from the tomato field at the University of Florida Gulf Coast Research and Education Center, Wimauma, FL, USA (39.6-m altitude, 27°45′23.7168″ N, 82°13′27.876″ W). The two soils were chosen in proximity to minimize broad-scale environmental variation, such as differences in climate, parent material, and overarching soil properties, allowing for a more direct comparison of land use effects.

Soils were mixed with 50% potting soil (Jolly Gardener Pro-Line HydraFiber HFC/B Growing Mix) to assist in moisture retention, given the sandy soil texture of Florida soils. The soil was mixed in a cement mixer to ensure homogeneity between the amendments and the soil before filling the pots. Fresh soil was collected before starting each experiment, with a 3-month interval between collections. Physical–chemical analysis was then conducted on the soil mixtures by the University of Florida Extension Soils Lab.

### Experimental design

Two in vivo greenhouse experiments were conducted, focusing on the influence of soil amendments and the increased *M. enterolobii* populations on plant health and soil microbiome dynamics.

The first experiment was conducted to evaluate the impact of various soil amendments on plant health, focusing particularly on their potential to influence the soil microbiome composition and mitigate disease susceptibility. We determined a standard inoculum of 5000 eggs per pot based on use in prior literature [13, 14]. This foundational experiment established a baseline understanding of how alterations in the soil environment could affect plant–microbe interactions and, by extension, plant health. Building upon the insights from this initial study, a more rigorous assessment was undertaken to determine the resilience and adaptability of the plant-associated microbiome under heightened inoculum pressure. To achieve this, we conducted a second experiment in which the disease challenge was escalated by introducing *M. enterolobii* eggs at a concentration 10 times greater than that used in the preliminary experiment (50,000 eggs). This experiment was run for 60 days, allowing for two infection cycles to maximize the clarity and significance of the microbial changes. In past work, it has been shown that an increased inoculum concentration of *Meloidogyne* spp. eggs can be linked to lower plant yield [15, 16]. This strategic amplification aimed to simulate a high pathogen load scenario, enabling the analysis of the core microbiome constituents that persist under such stress conditions and clarifying the microbiome’s role in plant disease resistance mechanisms. This escalated inoculum approach also stress-tests the previously observed disease-mitigating effects of soil amendments and uncovers the microbiome’s inherent capacity to buffer plants against intensified disease threats, thereby providing valuable insights into potential microbial-based strategies for enhancing plant resilience.

For each soil type, native soil (Na) and agricultural (Ag), there were four treatment groups, including a control, and eight replicates per treatment following a randomized complete block design. The four treatments were the control (C), nematode eggs (N), collagen + nematode eggs (Co), and chitin + nematode eggs (Ch). The high inoculum pressure experiment was conducted with the same treatment groups as previously described with five replicates per treatment.

### Treatment application

Five weeks after germination, tomato seedlings (cv. Little Napoli) were planted in 2-L plastic pots in a greenhouse (362 l m/ft^2^, 28 °C, and 65% rh). We fertilized the plants weekly (NPK 24–8–16) and hand-watered every 24 h.

Immediately before planting, collagen (Orgain, Amazon: B07BL69 CD2) and chitin (Thermo Scientific: AAJ6120636) were mixed in the soil using a cement mixer (as described previously). Collagen was incorporated at a concentration of 0.2% w/w [10], while chitin was added at a concentration of 1% w/w [11]. The same day, directly after transplanting the tomato seedlings, 3 small holes were made in the soil close to the root zone, and then a total of 5000 M*. enterolobii* eggs were applied equally divided among the 3 holes using a 1000-μL pipette. In the high inoculum pressure experiment, 50,000 M*. enterolobii* eggs were applied to the soil using the same method.

### Assessed plant- and nematode-related variables

For the first experiment, roots were collected 45 days after planting, while for the high inoculum pressure experiment, roots were collected after 60 days. In both cases, roots were separated from their respective shoots, a rhizosphere sample was taken, and the remaining soil was gently rinsed off. The plant growth responses, root fresh weight (RFW), and shoot dry weight (SDW) were measured. To measure the concentration of chlorophyll, the absorbances of chlorophyll A, chlorophyll B, and carotenoids were measured. A 5-mm leaf disk was cut from the third branch of each sample and placed in a 1.5-mL tube with 1 mL of 80% acetone. The samples were incubated at room temperature for 24 h, and then the absorbances were recorded. The resulting data were analyzed using the formulas described by Lichtenthaler and Wellburn [17, 18] (Table 1).

**Table 1:** Formulas for the calculation of pigments (Lichtenthaler and Wellburn, 1983)

Chlorophyll a	12.7 (A663) − 2.69 (A646)
Chlorophyll b	22.9 (A646) − 4.68 (A663)
Carotenoids	(1000 A470 − 3.27[Chl a] − 104 [Chl b])/227
Total chlorophyll	20.2 (A646) + 8.02 (A663)
Total pigments	Chlorophyll a + chlorophyll b + carotenoids

Then the gall index for roots was assessed using two indices rated on a 0 to 5 scale, based on the number of egg masses and galls. In the Brito et al. index, 0 = 0 egg masses and galls per root system; 1 = 1–2 egg masses and galls per root system; 2 = 3–10 egg masses and galls per root system; 3 = 11–30 egg masses and galls per root system; 4 = 31–99 egg masses and galls per root system; and 5 = ≥ 100 egg masses and galls per root system [14]. In the Hussey and Janssen index, 0 = 0% egg masses and galls per root system; 1 = few or trace amounts of galls present in a root system; 2 = < 25% of the root system is galled, 3 = 26–50% of the root system is galled; 4 = 51–75% of the root system is galled; and 5 = > 75% of the root system is galled [19].

For nematode egg extraction, for both inoculum collection and egg quantification, we used the J. A. Brito, J. Desaeger, and D. W. Dickson method [14]. We extracted eggs from the roots using 1% NaOCl. The NaOCl solution was added until the liquid just fully covered the roots. The root-NaOCl mixture was blended on the highest setting for approximately 30 s, and the blended remains were added to a series of sieves (No. 200 and No. 500 mesh, respectively). The nematode suspension was rinsed with a continual stream of tap water until there was no visible trace of bleach on the sieves. The eggs caught by the No. 500 sieve were then resuspended in water to a final concentration of 1000 eggs mL^−1^.

### Rhizosphere isolation and DNA extraction

Soil samples for 16S rRNA gene and ITS sequencing were extracted from the soil rhizosphere (collected prior to root washing), following the protocol from T. Simmons, D. F. Caddell, S. Deng, and D. Coleman-Derr [20]. First, 5 g of roots was removed with the soil still attached and placed into 25 mL of epiphyte removal buffer [20]. The tubes were then placed in an ultrasonic bath for 10 min at room temperature to detach microbes from the roots. Roots were then removed, and the remaining solution was centrifuged for 10 min at maximum speed. The buffer was then discarded, and the remaining rhizosphere was used for DNA analysis.

The DNA was extracted from 250 mg of soil using the Zymo Quick-DNA™ Fecal/Soil Microbe Miniprep Kit (Zymo Research, Irvine, CA, USA). DNA purity was assessed using spectrophotometry (NanoDrop model, Thermo Fisher, Waltham, MA, USA). DNA was sequenced for ITS and 16S analysis at SeqCenter in Pittsburgh, PA, USA. The samples were prepared for sequencing using the Zymo Quick-16S Plus or Quick-ITS Plus Library Prep kits with forward and reverse primers (Table 2) and unique dual indexes. Pooled PCR products were cleaned up using the ZymoBIOMICS DNA Clean & Concentrator Kit to remove unwanted contaminants. Following cleanup, the pool of samples was sequenced on a P1 600cyc NextSeq 2000 flow cell generating 2 × 301-bp paired-end reads. Quality control and adapter trimming were performed with bcl-convert1 (v4.2.4). The rarefaction curves and other sequencing quality control data were included as Supplementary Fig. 1.

**Table 2:** Target region to be used in amplicon sequencing for fungal and bacterial microbial communities

Target	Region	Sequences
Bacterial community	16S (V3–V4)	Forward (5′-CCTACGGGDGGCWGCAG-3′) Reverse (5′-GACTACHVGGGTATCTAATCC-3′)
Fungal community	ITS2 (ITS3f, ITS4r)	Forward (5′-GCATCGATGAAGAACGCAG-3′) Reverse (5′-TCCTCCGCTTATTGATATGC-3′)

### Ultra-performance liquid chromatography-tandem mass spectrometry (UPLC-MS/MS) for metabolite analysis

With the goal of identifying and quantifying the metabolites present in soil samples without prior knowledge of what these metabolites might be, an untargeted soil metabolomic analysis was conducted following the procedures of [21]. On the last day of each experiment, for both high and standard inoculation pressure, 2 g of rhizosphere was collected and mixed with 10 mL of deionized water. The mixture was centrifuged for 5 min at 12,300 × g, and the supernatant was collected in a new tube, which was directly stored at − 80 °C until use. The frozen samples were thawed, vortexed for 30 s, and centrifuged at 1110 × g for 3 min, and 6 mL of the supernatant was placed in the corresponding numbered 50-mL centrifuge tube, frozen at − 80° C overnight, and vacuum freeze-dried. A volume of 500 μL of 70% methanol internal standard extract was added into each tube, vortexed for 15 min, and sonicated in an ice water bath (cat. no. KQ5200E) for 10 min. Tubes were centrifuged at 4 °C for 3 min at 12,300 × g; the supernatant was filtered through a 0.22-μm pore size membrane and stored in the injection vial for LC–MS/MS detection. Metabolites were identified and quantified using the database from Metware Biotechnology Inc., Ltd. in Woburn, MA, USA. Variable importance in projection (VIP) was calculated using orthogonal partial least squares discriminant analysis (OPLS-DA) to identify metabolite variation among samples.

### Data analysis and statistical design

A *t*-test was applied for comparison between pigments, egg number, gall counts, and comparison among biomass responses, dry shoot weight, and root fresh weight. For all analyses, the assumption of normality was checked by Shapiro–Wilk and Kolmogorov–Smirnov tests prior to analysis. The Kruskal–Wallis one-way analysis of variance on ranks (*P* < 0.05) was applied for significant means when normality was not met. SigmaPlot® version 14.5 was used for statistical analyses.

SHAMAN database online version available at https://shaman.pasteur.fr/ was used to process and assign taxa to raw fastq.gz files [21]. Sequences were clustered based on the operational taxonomic unit (OTU) at a threshold of 97% similarity. Taxonomy assignment was performed using the SILVA database for bacterial sequences and the UNITE database for fungal sequences [22]. The relative abundances, as well as Alpha and Inverse Simpson diversity indexes, were calculated according to the default parameters of Volant et al. [23]. Raw read data was submitted to the NCBI SRA under the accession number PRJNA1120455. The accession numbers for the sequences are SRX24815206 to SRX25586782. After taxonomic assignment, microbial networks were constructed in R v4.3.1 [24] using methods adapted from Poudel et al. [25]. Bacterial and fungal OTUs were assessed to determine their co-occurrence with desired plant phenotypes, leveraging a phenotype-OTU (PhONA) network analysis. To initiate this analysis, associations between bacterial and fungal OTUs and disease severity (the phenotype) were evaluated following infection by *M. enterolobii* in tomato plants cultivated in each of the soil treatments. This association was established by identifying treatments that fell within the bottom quartile of eggs per gram of roots. Logistic regression was used to establish the log odds of associations between the top 5% most abundant OTUs and the phenotype, thus facilitating an understanding of how the presence or absence of each OTU correlates with the phenotype. OTUs for which *P* < 0.05 were then represented in a network format using the igraph package in R [26].

In the OTU-OTU network analysis, positive and negative relationships were identified within the microbial community. A table of OTU relative abundance across the soil samples was analyzed. Spearman’s rank correlations were evaluated pairwise for OTU relative abundances, using a significance threshold of *P* < 0.001. These correlations were used to assemble an adjacency matrix, with the corresponding network representation plotted using igraph, with nodes representing OTUs and edges denoting significant correlations.

The core microbiome was assessed for bacterial and fungal OTUs, with core microbiomes determined by comparing the experimental treatments soil type (native vs. agricultural soil) and experiment (high vs. standard inoculation). This assessment was made using the microbiome R package [27]. To determine the core microbiome, an abundance-occurrence approach was taken, defining the “core” as OTUs found between groups at a 0.001 abundance detection limit and a 50% prevalence [28].

## Results

For both experiments, agricultural soil had higher nutrient levels compared to native soil, with few exceptions (Table 3), as measured prior to the start of the experiments.

**Table 3:** Soil chemical parameters assessment (organic matter content, macro- and micronutrients) for the two types of soils used in this study (mg kg^−1^ in the soil)

Soil type	Cu2 +	Mn2 +	Zn2 +	Ca2 +	Mg2 +	K +	P3-	Organic matter %	pH
High inoculum pressure exp									
Native soil	10.98	7.85	8.58	579.74	143.32	45.19	146.49	1.86	6.36
Agricultural soil	16.34	9.42	9.42	628.47	163.72	75.41	182.72	3.24	6.22
Standard inoculation exp									
Native soil	12.36	10.20	9.77	858.98	142.01	53.74	171.54	2.62	6.50
Agricultural soil	16.80	10.68	9.78	676.83	102.44	49.52	194.46	2.01	6.24

A slight decrease in gall count was observed when amendments were added to the soil when compared to the control with nematode inoculated (Table 4). However, a statistical difference was only found for the Hussey and Janssen (2002) metrics.

**Table 4:** Gall indices assessed at harvest from the roots of tomato cv. Little Napoli plants under standard (5000 eggs) and high inoculation pressure (50,000 eggs). The Student’s *T*-test was performed between chitin + N or collagen + N treatments and infected control treatment (*N* = nematode)

	Native soil				Agricultural soil			
Treatment	Uninfected control	Infected control	Chitin + N^a^	Collagen + N	Uninfected control	Infected control	Chitin + N	Collagen + N
Brito et al., 2020 [14]								
Standard inoculation	1.75***	5	4.87 ns	4.75 ns	0***	5	5 ns	4.625 ns
High inoculum pressure	1.2**	4.8	4.6 ns	4.8 ns	1.6*	5	5 ns	5 ns
Hussey & Janssen, 2002								
Standard inoculation	0.625***	4.375	3.375*	2.625***	0***	3.75	3.125 ns	2.375**
High inoculum pressure	0.6**	4.4	4.2 ns	3.6 ns	0.8**	4.8	5 ns	3.6**

^a^*N* = (nematode) *M. enterolobii*. Statistical significance is indicated by *P < 0.05, **P < 0.01, ***P < 0.001, *ns* not significant

**Fig. 1:**
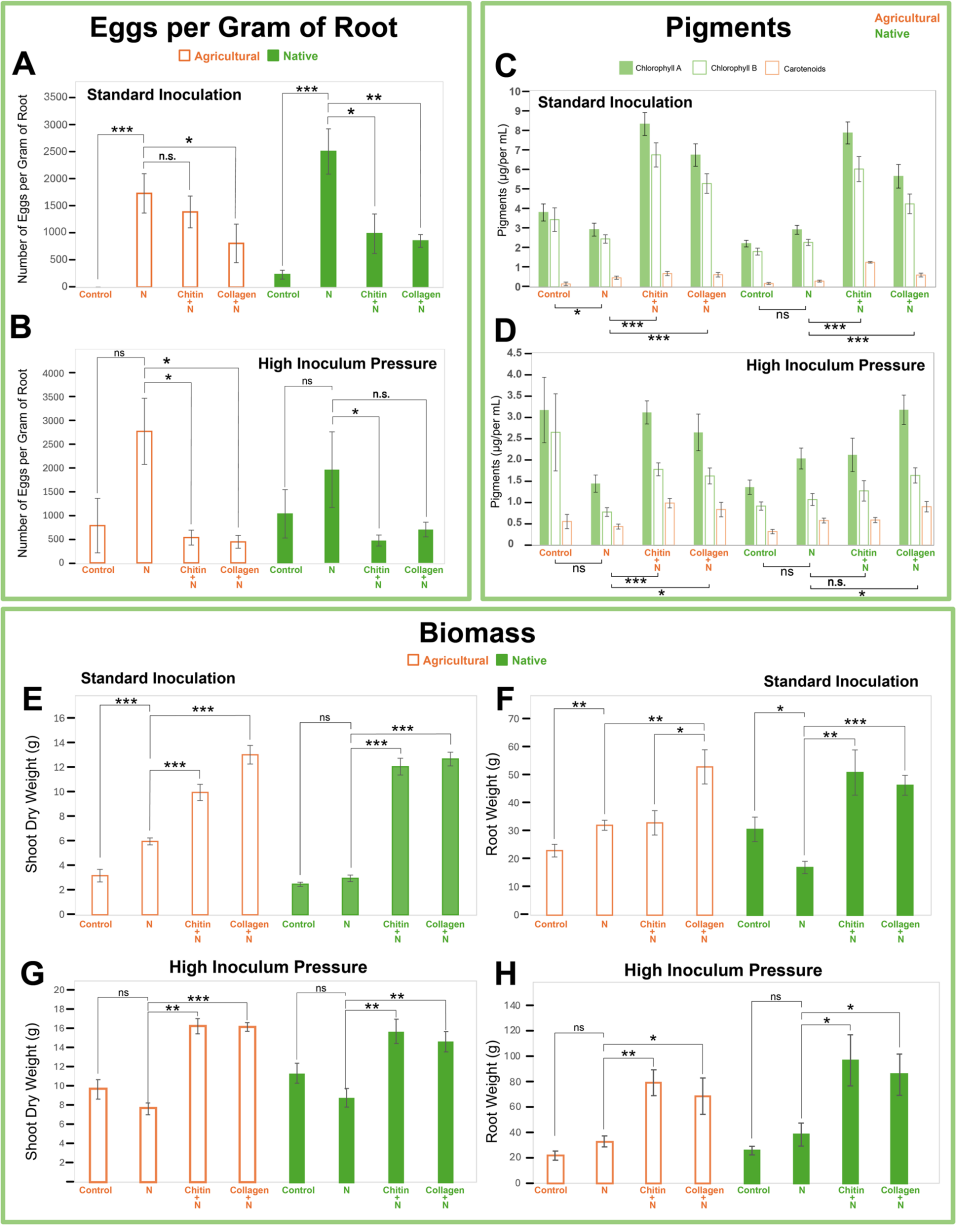
Comparison of plant health parameters and *M. enterolobii* infection in tomato cv. Little Napoli. Number of *M. enterolobii* eggs per gram of root, measured at 45 days (**A**) and 60 days (**B**) after inoculation, respectively, showing responses under standard (5000 eggs) and high inoculation pressure (50,000 eggs). Photosynthetic pigments in leaves under standard (**C**) and high inoculation (**D**). Biomass parameters with dry shoot (**E**) and root (**F**) weights under standard inoculation; Dry shoot (**G**) and root (**H**) weights at high inoculum pressure. *Significant at the 0.05 probability level by Mann–Whitney rank-sum test (**A**, **B**) and Student’s *T*-test (**C**, **D**, **E**, **F**, **G**, **H**). N = (nematode) *M. enterolobii*. Statistical significance is indicated by *P < 0.05, **P < 0.01, ***P < 0.001, ns not significant. Error bars denote ± SE. Sample sizes are eight replicates for standard inoculation and five for high inoculum pressure

Regarding egg count, nematode infection was observed to be significantly lower in the native soil with the addition of either soil amendment (Fig. 1A).

We observed a decrease in *M. enterolobii* egg density (eggs per gram of root) in the collagen- and chitin-amended treatments. However, this reduction appears to be largely driven by an increase in root biomass promoted by these amendments, rather than direct suppression of nematode reproduction. The addition of chitin to the soil reduced egg number by 60% (*P* < 0.01) and collagen reduced eggs per gram of root by 66% compared to the inoculated control (*P* < 0.001). In the agricultural soil, the number of eggs per gram of roots decreased in soil amended with collagen (*P* < 0.05); however, we observed a non-statistically significant reduction for chitin-amended soil compared to the inoculated control (*P* = 0.67). Under high inoculum pressure, the addition of chitin and collagen in the agricultural soil led to a significant reduction in the number of eggs per gram of root by 81% and 84%, respectively (*P* < 0.01), compared to the inoculated control (Fig. 1B). In native soil, the amendments also resulted in a decrease in the number of eggs, with chitin-amended soils showing a 76% (*P* = 0.03) reduction and collagen-amended soils showing a statistically insignificant 64% reduction compared to the inoculated control (*P* = 0.15).

Chlorophyll content also increased, suggesting an effect on plant physiology, with collagen and chitin treatments overall boosting chlorophyll by 100% and 170%, respectively (*P* < 0.001) compared to the inoculated control (Fig. 1C). Similar trends were observed under high inoculum pressure, with amended agricultural soil showing increases in pigment concentration compared to the inoculated control (Fig. 1D). However, in native soil, the chitin group did not significantly increase pigment content compared to the infected control. Furthermore, tomato plants displayed a lower overall pigment concentration compared to the standard inoculation experiment.

With the addition of chitin in the agricultural soil, dry shoot weight increased by 66.72% (*P* < 0.01) when compared to the nematode-infected control (Fig. 1E). Similarly, adding collagen to the agricultural soil yielded a 120% increase in dry weight compared to the inoculated control (*P* < 0.001). In native soil, the addition of chitin and collagen yielded a 310% increase (*P* < 0.01) and a 330% increase in dry weight increase (*P* < 0.001) compared to the inoculated control, respectively (Fig. 1E). Introducing collagen to agricultural soil increased root weight by 64.94% (*P* < 0.01) compared to the inoculated control (Fig. 1F). Additionally, agricultural soil amended with collagen displayed a 60.65% (*P* < 0.05) greater increase in root weight than soils amended with chitin compared to the inoculated control. Adding chitin to agricultural soil also yielded a root weight increase of 2.67% compared to the inoculated control (*P* = 0.86). In native soil, adding chitin yielded a 200% increase in root weight (*P* < 0.001), while adding collagen yielded a 170% increase in root weight compared to the inoculated control (*P* < 0.01).

Under high inoculum pressure, agricultural soil also produced increased dry shoot weight, specifically by 110% (*P* < 0.001) with chitin amendment and by 110% (*P* < 0.001) with collagen compared to the inoculated control (Fig. 1G). In native soil, the chitin treatment produced a 78% increase (*P* < 0.01), while collagen produced a 65.5% increase (*P* < 0.01) compared to the unamended, infected treatment. The roots in agricultural soil amended with collagen exhibited a 110% increase, while those with a chitin amendment showed a 140% increase compared to the inoculated control (Fig. 1H). In native soils, chitin produced a 150% increase and collagen a 120% increase in root biomass compared to the inoculated control.

**Fig. 2:**
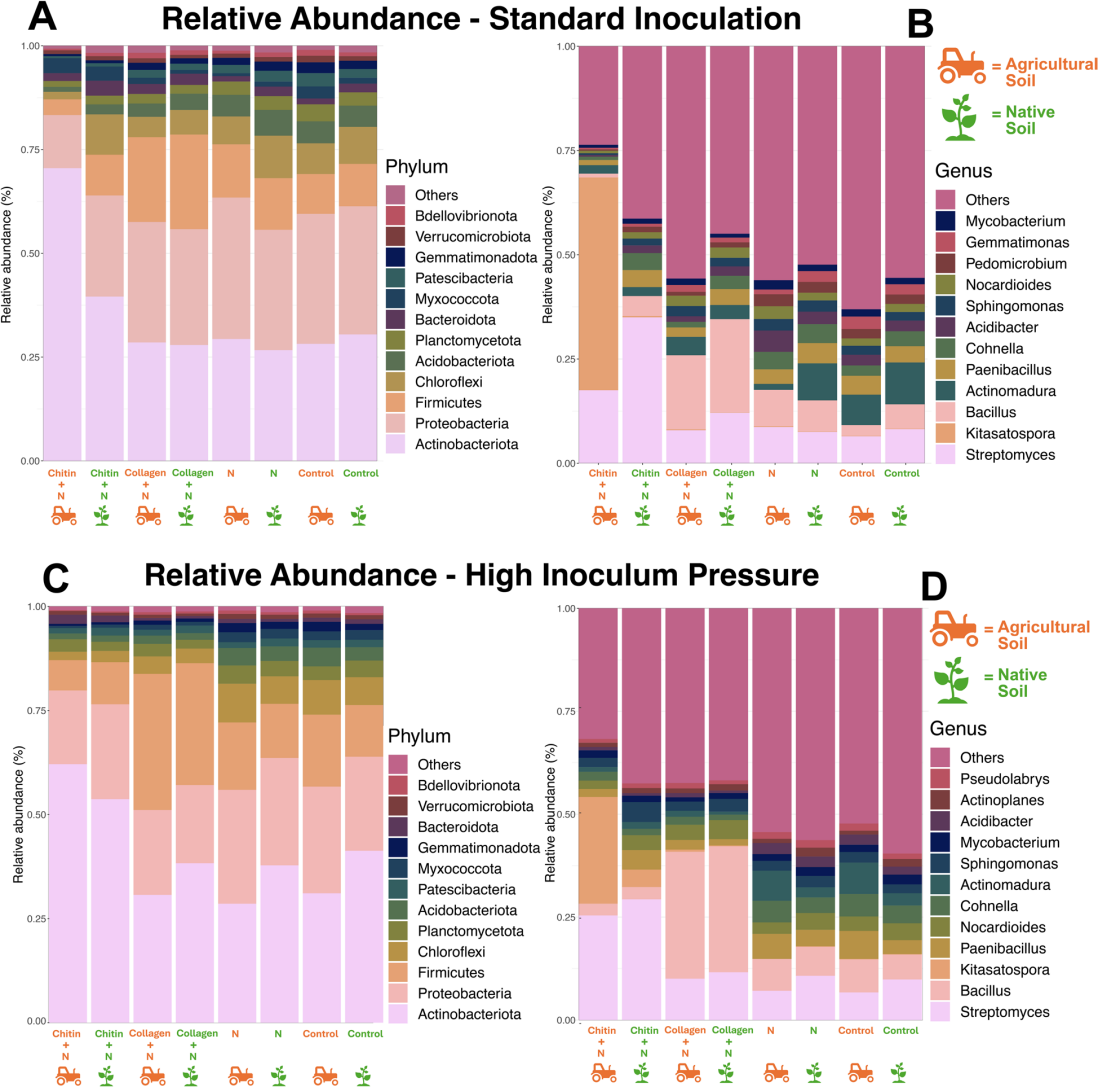
Relative abundance of operational taxonomic units (OTUs) of the 12 most abundant bacteria. Bacteria are displayed at the **A**, **C** phylum and **B**, **D** genus level. Bacterial community samples were taken from tomato cv. Little Napoli rhizosphere at the time of harvest. Comparison of standard inoculation (5000 eggs) (**A**, **B**) and high inoculum pressure (50,000 eggs) (**C**, **D**). N = (nematode) *M. enterolobii*. Means of five replicates

The uptick in biomass and photosynthetic pigments, alongside a reduction in nematode eggs, coincided with an alteration in the microbial community. However, this reduction appears to be due to the amendments enhancing plant growth, effectively diluting egg density per unit of root tissue. In chitin-treated soils compared to nematode-treated soils, there was a notable increase in the relative abundance of phyla such as *Actinobacteria* (*P* = 0.008 and *P* < 0.001, respectively, for standard and high inoculum pressure) (Fig. 2A) (Supplementary Table 1).

Moreover, the genus *Kitasatospora* has a particularly relative high abundance in agricultural soil treated with chitin, whereas native soil treated with chitin exhibits an increase in *Streptomyces* compared to other treatment groups (Fig. 2B) (Supplementary Table 2). At the genus level, the collagen-treated group in both soil types displayed a pronounced increase in the genus *Bacillus* compared to other treatments. Similar results were observed in the experiment under high inoculum pressure (Fig. 2C, D). In the agricultural soil treated with chitin, *Kitasatospora* dominated, whereas the collagen treatment resulted in a similar increase in the *Bacillus* population. However, the *Bacillus* population was higher in the high-pressure experiment compared to the standard.

**Fig. 3:**
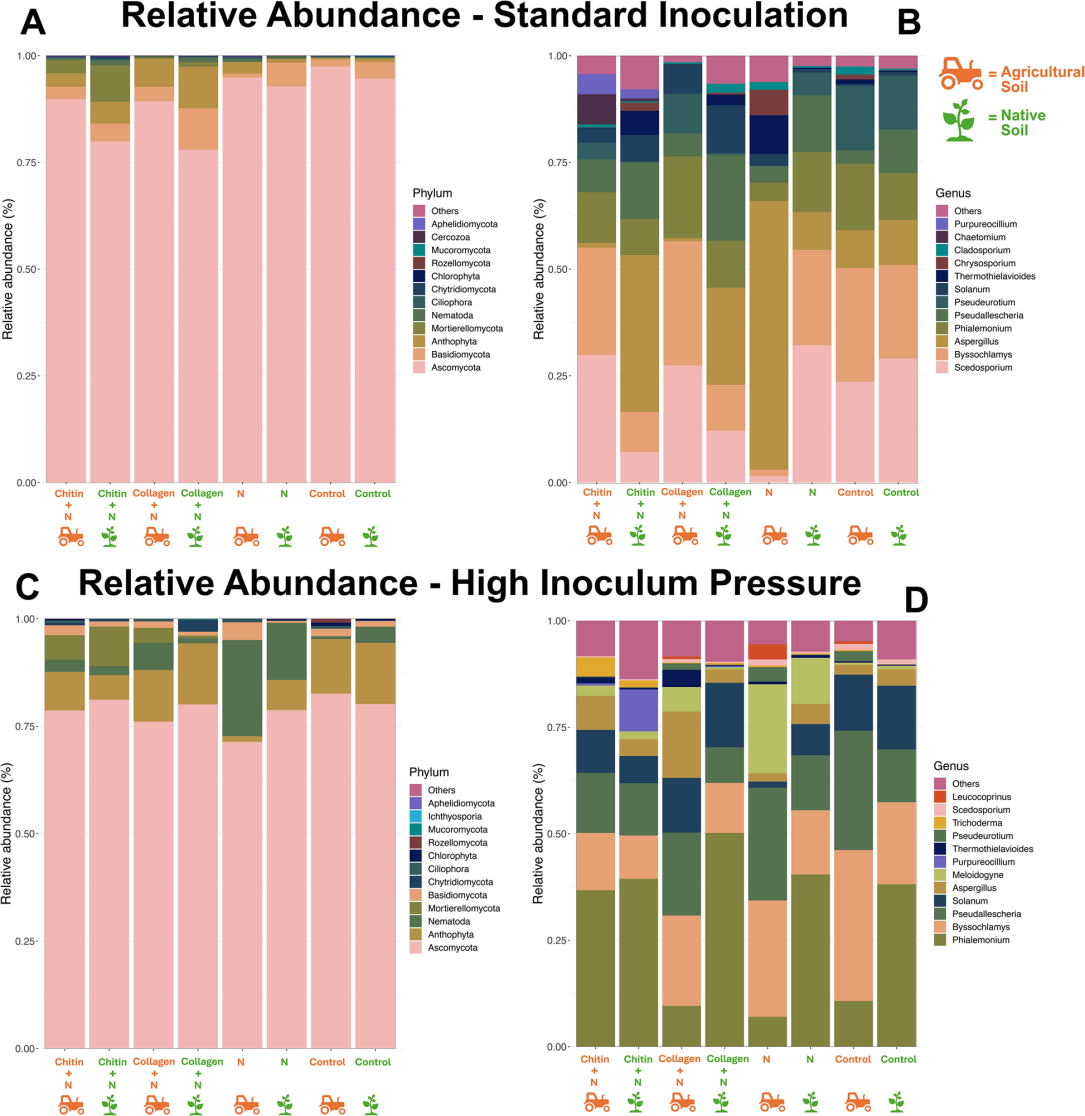
Relative abundance exhibiting the 12 most abundant fungal microbial assemblages. Fungal microbes are displayed at the phylum (**A**, **C**) and genus (**B**, **D**) level. Fungal community samples are taken from tomato cv. Little Napoli rhizosphere at the time of harvest. Comparison of standard inoculation (5000 eggs) (**A**,** B**) and high inoculum pressure (50,000 eggs) (**C, D**). N = (nematode) *M. enterolobii*. Means of five replicates

ITS sequencing revealed shifts in the abundance of fungi in response to experimental treatment groups (Supplementary Tables 3 and 4). Considering phylum distribution, the standard inoculation collagen-treated groups exhibited an elevation in Anthophyta for both soil types (Fig. 3A).

In contrast, the chitin-treated groups showed a rise in Mortierellomycota compared to other groups. While native soil generally presented a higher abundance of Basidiomycota, the collagen-treated group in native soil surpassed this, boasting the highest abundance. Differences in genus were observed between amended soil types, with amended agricultural soil containing higher levels of *Byssochlamys* and *Scedosporium*, while the amended native soil showed a higher abundance of *Aspergillus* and *Pseudallescheria* (Fig. 3B). The group under high inoculum pressure showed a similar trend with more alignment under soil type than specific treatment (Fig. 3D). This group also showed an overall increase in Anthophyta, with the most abundant groups being the control and collagen-treated groups (Fig. 3C). An increase in the phylum Nematoda was also seen, but most dramatically in the infected control groups for both soil types, corresponding with an increase in *Meloidogyne*. In the high inoculum group, the chitin amendment led to an increase in the *Phialemonium* spp. compared to the other treatments in the agricultural soil (Fig. 3D).

**Fig. 4:**
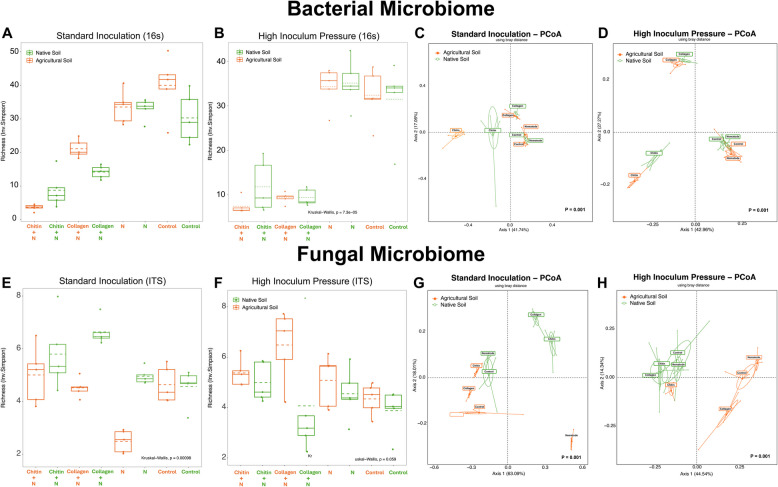
Visualization of microorganism diversity in tomato cv. Little Napoli rhizosphere. Bacterial (**A**, **B**) and fungal (**E**, **F**) diversity under **A**, **E** standard (5000 eggs) and **B**, **F** high inoculum pressure (50,000 eggs) through Inverse Simpson Plot. Principal coordinates analysis (PCoA) based on Bray–Curtis distance metric of bacterial (**C**, **D**) and fungal (**G**, **H**) diversity under **C**, **G** standard and **D**, **H** high inoculum pressure. N = (nematode) *M. enterolobii*. The percentage of the variation explained by the plotted principal coordinates is indicated on the axes. Color indicates the treatment group, as indicated in the key. In the experiment under high inoculum pressure, a similar decrease in diversity was observed in the amended groups; however, a stronger decrease was observed in the collagen-amended groups (**B**). No clear distinction was observed in the ITS data

Along with these shifts in the amended soils, there was a decrease in diversity compared to the unamended groups, with chitin showing the lowest richness (Fig. 4A).

In the experiment under high inoculum pressure, a similar decrease in diversity was observed in the amended groups; however, a stronger decrease was observed in the collagen-amended groups (Fig. 4B). No clear distinction was observed in the ITS data.

The microbiome profiles of the treatment groups showed statistically significant separation in diversity (permutational multivariate ANOVA [PERMANOVA], *P* = 0.001; Fig. 4C, D, G, H). In the bacterial microbiome, the amended groups demonstrated clear differentiation from each other and from the non-amended groups. The pronounced separation between treatment groups is primarily attributed to the highest principal coordinate 1 (*PC1* = 47.74%). Under high inoculum pressure, the differentiation between groups is more distinct, with the collagen and chitin groups isolated from the unamended groups, attributed to the highest principal coordinate 1 (*PC1* = 42.96%). In the fungal microbiome, a separation was not seen based on the treatment type but instead by soil type (Fig. 4H).

The bacterial OTU-OTU networks revealed changes in the bacterial microbiome in response to soil type, inoculation intensity, and soil amendment. In the native soil under standard inoculation, amendments in the soil increased organisms such as *Streptomyces* Sd-13 and *Bacillus* spp. (Fig. 6G, H).

**Fig. 5:**
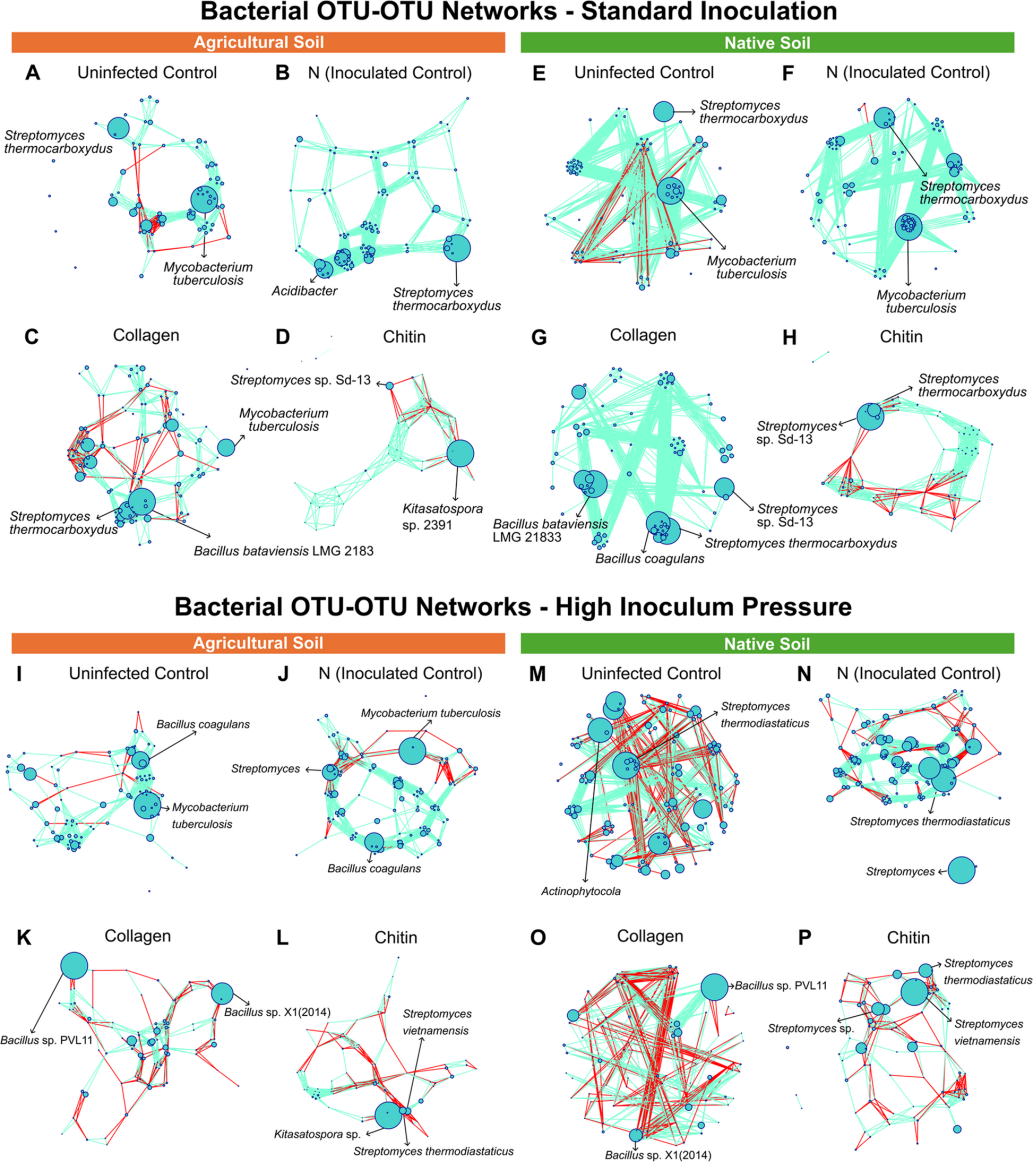
16S rRNA gene OTU-OTU network constructed from tomato (cv. Little Napoli) rhizosphere samples. Samples were taken from agricultural (**A**, **B**, **C**,** D**; **I**, **J**, **K**, **L**) and native (**E**, **F**, **G**, **H**; **M**, **N**, **O**, **P**) soil under standard (5000 eggs) (**A**, **B**, **C**, **D**, **E**, **F**, **G**, **H**) and high (50,000 eggs) (**I**, **J**, **K**, **L**, **M**, **N**, **O**, **P**) inoculation. Node size is proportional to relative abundance, and edge color indicates positive (blue) and negative (red) correlations for the four treatments: negative control, inoculated control (nematode), collagen-treated soil (Co) and chitin-treated soil (Ch). N = (nematode) *M. enterolobii* (five replicates per treatment)

A similar increase in the relative abundance of *Bacillus* and *Streptomyces* groups were observed in the agricultural soil; however, in the group treated with chitin amendment, there was a notable shift towards *Kitasatospora* sp. 2391 emerging as the predominant organism (Fig. 5D). Under high inoculum pressure, similar outcomes were observed, demonstrating an increase in various *Bacillus* spp. in soils amended with collagen (Fig. 5K, O). Additionally, in the agricultural soil, a rise in *Kitasatospora* was also noted, mirroring the trend seen in the standard inoculation group (Fig. 5L).

**Fig. 6:**
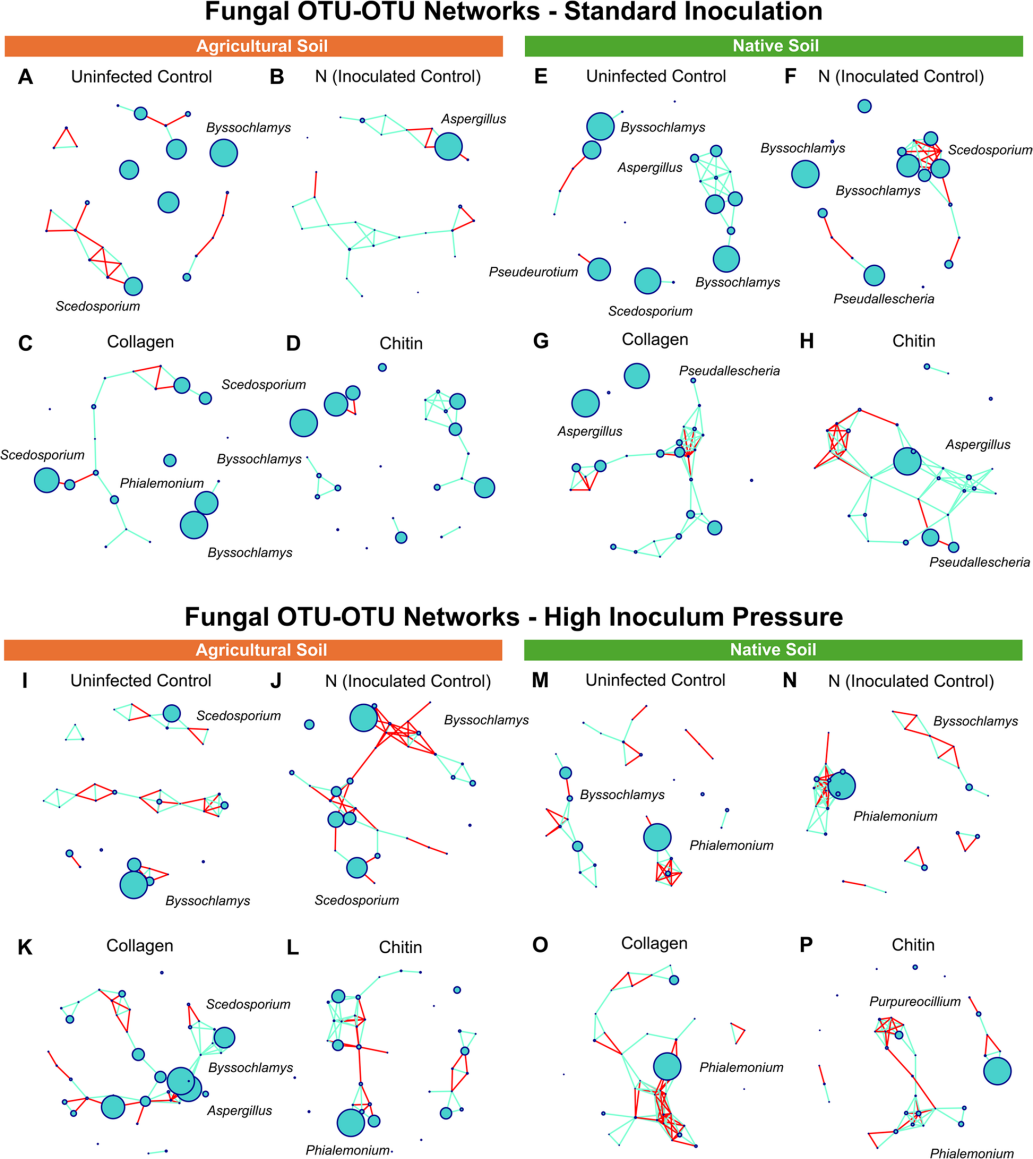
ITS OTU-OTU network constructed from tomato (cv. Little Napoli) rhizosphere samples. Samples were taken from agricultural (**A**, **B**, **C**, **D**; **I**, **J**, **K**, **L**) and native (**E**, **F**, **G**, **H**; **M**, **N**, **O**, **P**) soil under standard (5000 eggs) (**A**-**H**) and high (50,000 eggs) (**I**, **J**, **K**, **L**, **M**, **N**, **O**, **P**) inoculation. The node sizes correspond to the relative abundance, and edge color corresponds to positive (blue) and negative (red) correlations for the four treatments: negative control, inoculated control (nematode), collagen-treated soil (Co), and chitin-treated soil (Ch). N = (nematode) *M. enterolobii* (five replicates per treatment)

In the fungal OTU-OTU networks, changes in relative abundance were less notable compared to the bacterial networks, as many genera remained consistent across different treatments (Fig. 6).

However, in the native soil under high inoculum pressure, *Phialemonium* emerged as the dominant organism (Fig. 6M, N, O, P). This genus also specifically dominated the chitin group in the agricultural soil under similar conditions (Fig. 6L).

Regarding overall network dynamics, more negative edges were observed under high inoculum pressure compared to standard inoculation scenarios, indicating the potential for more competitive interactions between microbes (Table 5).

**Table 5:** Summary of key network metrics for bacterial community analysis

Treatment	Positive edges	Negative edges	Total connections	Average node degree	Number of nodes
Standard inoculation agricultural soil					
Uninfected control	334	46	380	10.56	72
Inoculated (nematode)	1098	0	1098	22.875	96
Collagen + N*	421	124	545	10.80	101
Chitin + N	163	29	192	9.37	41
Standard inoculation native soil					
Uninfected control	229	12	241	5.95	81
Inoculated (nematode)	1339	2	1341	27.65	97
Collagen + N	877	0	877	21.13	83
Chitin + N	469	71	540	14.80	73
High inoculum pressure agricultural oil					
Uninfected control	492	17	509	11.31	90
Inoculated (nematode)	627	93	720	13.46	107
Collagen + N	241	128	369	8.11	91
Chitin + N	235	112	347	8.90	78
High inoculum pressure native soil					
Uninfected control	616	292	908	14.76	123
Inoculated (nematode)	962	108	1070	15.97	134
Collagen + N	350	214	564	12	94
Chitin + N	182	132	314	8.05	78

^*^N = (nematode) *M. enterolobii*

Key network metrics varied among treatment groups, highlighted in Table 5, indicate distinct microbial community responses.

In the fungal microbial networks, the high inoculum pressure groups also resulted in more negative edges and a competitive microbial environment (Table 6).

**Table 6:** Summary of key network metrics for fungal community analysis

Treatment	Positive edges	Negative edges	Total connections	Average node degree	Number of nodes
Standard inoculation agricultural soil					
Uninfected control	9	16	25	2	25
Inoculated (nematode)	27	7	34	2.43	28
Collagen + N*	13	5	18	1.71	21
Chitin + N	22	2	24	1.85	26
Standard inoculation native soil					
Uninfected control	24	3	27	2.57	21
Inoculated (nematode)	14	14	28	3.11	18
Collagen + N	45	12	57	3.93	29
Chitin + N	52	16	68	4.53	30
High inoculum pressure agricultural soil					
Uninfected control	33	23	56	2.95	38
Inoculated (nematode)	20	35	55	3.44	32
Collagen + N	56	22	78	3.80	41
Chitin + N	39	20	59	3.28	36
High inoculum pressure native soil					
Uninfected control	24	20	44	2.67	33
Inoculated (nematode)	36	30	66	4.26	31
Collagen + N	47	40	87	4.70	37
Chitin + N	33	24	57	3.35	34

^*^N = (nematode) *M. enterolobii*

**Fig. 7:**
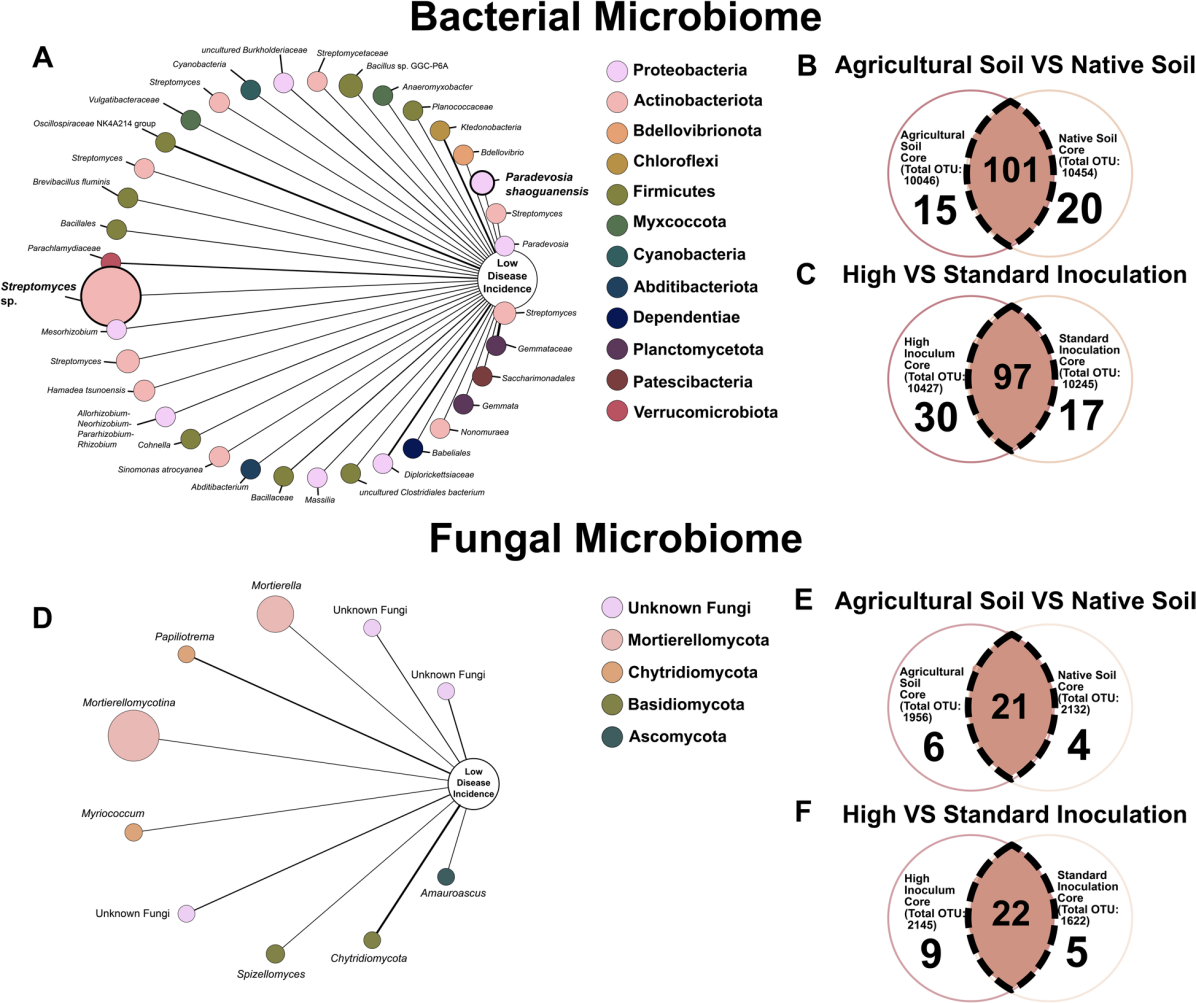
The OTU-phenotype network and core microbiome. This network uncovers **A** 36 and **B** 10 significant OTUs (operational taxonomic units) (*P* < 0.05) strongly associated with the log odds probability, as determined by logistic regression analysis, of decreased disease resistance in plants infected with *M. enterolobii*. This association was established by considering treatments falling within the bottom quartile of eggs per root gram. Nodes in the network are color-coded according to the corresponding phylum of the OTU, while node size reflects its relative abundance. The width of edges signifies the strength of the probability coefficient, with solid lines indicating positive associations. Additionally, a bolded font and node indicate members of the core microbiome. Bacterial (**B**, **C**) and fungal (**E**, **F**) Venn diagrams displaying the number of OTUs of soil types (**B**, **E**) and experiment (**C**, **F**) found and the core microbiome between them at a 0.001 relative abundance detection limit and a 50% prevalence

The bacterial phenotype-OTU network indicates OTUs significantly (*P* < 0.05) associated with lower disease severity (Fig. 7A, D).

Two OTUs, *Paradevosia shaoguanensis* and *Streptomyces* sp*.*, were identified as both associated with lower disease incidence and a part of the overall core microbiome. A phenotype-OTU analysis was performed on the ITS data, revealing 10 significant associations (*P* < 0.05) with low disease incidence (Fig. 7D). These organisms encompassed four different phyla, including three unidentified fungi. Mortierellomycotina was found in the highest abundance, while the strongest coefficient was attributed to Chytridiomycota.

A core microbiome across soil types was also identified, comprising 101 OTUs in the 16 s data and 21 in the ITS data at a 0.001 abundance detection limit and 50% prevalence (Fig. 7B, E). Between experiments, 97 bacterial OTUs and 22 fungal OTUs were found to compose the core microbiome shared between standard inoculation and high inoculum pressure under the same parameters (Fig. 7C, F).

**Fig. 8:**
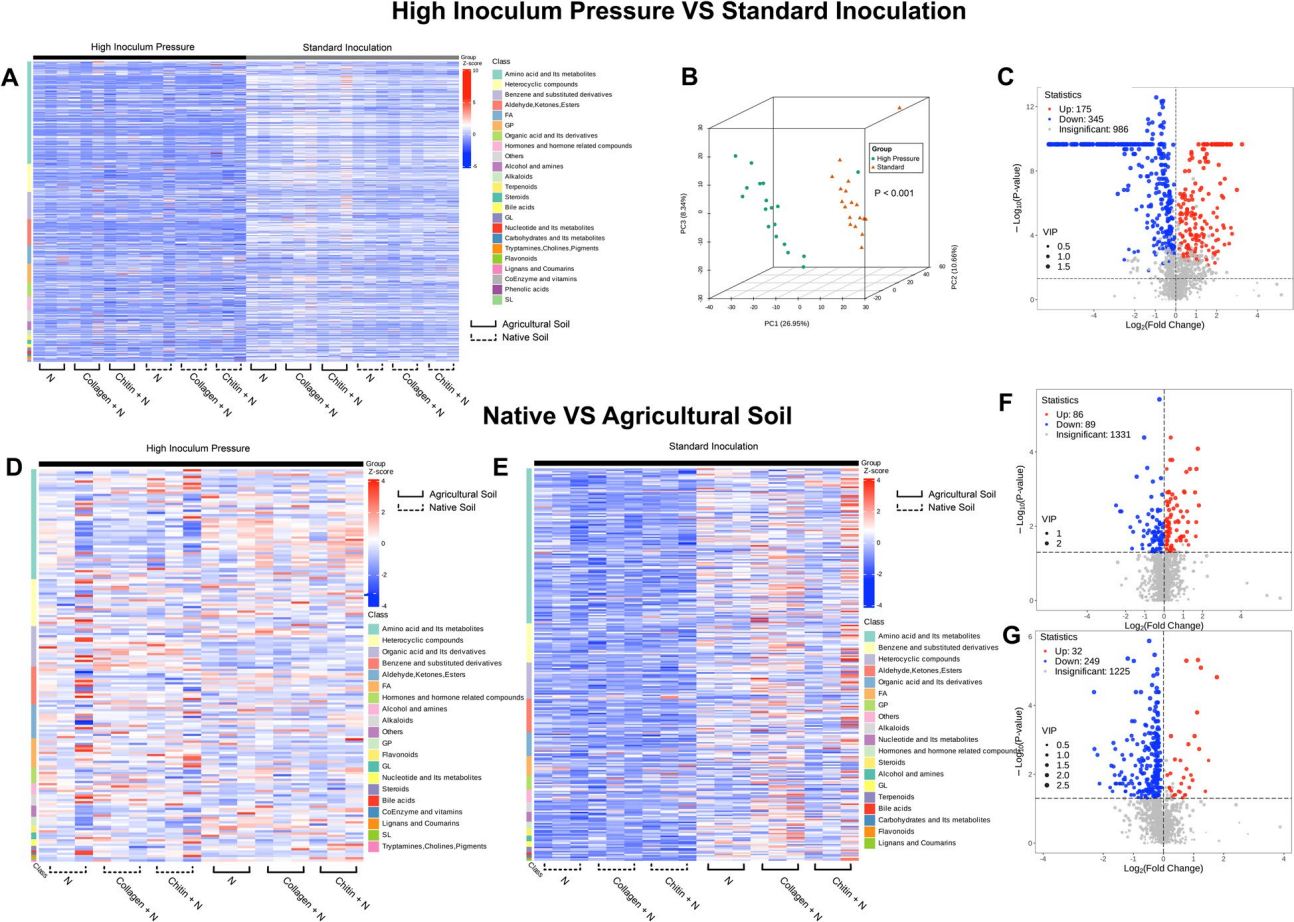
Untargeted soil metabolomics: differential analysis between high inoculum pressure and standard inoculation conditions. This figure contains comparisons between (*ABC*) two nematode inoculation levels and (*DEFG*) two soil types. The analyses include the following: **A** A heatmap displaying the expression levels of various metabolic groups. **B** A principal coordinates analysis (PCoA) illustrating the clustering of metabolic profiles. **C** A volcano plot that identifies significantly upregulated and downregulated metabolites. **D** A heatmap displaying the expression levels of various metabolic groups under high inoculum pressure (50,000 eggs). **E** A heatmap displaying the expression levels of various metabolic groups under standard inoculation (5000 eggs. **F** A volcano plot identifying significantly upregulated and downregulated metabolites under high inoculum pressure. **G** A volcano plot identifying significantly upregulated and downregulated metabolites under standard inoculation

Untargeted soil metabolomic analysis, a comprehensive approach used to identify and quantify a wide range of metabolites present in soil samples without prior knowledge of what these metabolites might be, was conducted, revealing a notable disparity between high inoculum pressure and standard inoculation. The high inoculum pressure group exhibited lower expression of metabolic groups (classes), whereas the standard inoculation group showed increased expression (Fig. 8A, C).

Additionally, in the PCoA analysis of metabolites, distinct separation between the experiments was observed. The pronounced segregation between treatment groups is primarily attributed to the highest principal coordinate 1 (*PC1* = 26.95%) (Fig. 8B). The most prominent metabolic group was amino acids and their metabolites, likely indicating higher activity levels in the soil.

In comparing untargeted metabolomics under standard inoculation across soil types, a visible difference was evident, with decreased expression observed in the native soil, contrasting with elevated expression in agricultural soil (Fig. 8E). However, this contrast was not evident under conditions of high inoculum pressure. While metabolite expression remained similar in the agricultural soil under both conditions, the native soil exhibited heightened expression levels under high inoculum pressure as well (Fig. 8D). This trend was further illustrated in the volcano plots, where numerous metabolites were down-expressed in the standard inoculation group (Fig. 8G), while the high inoculum pressure group showed a more balanced distribution of up- and down-expressed metabolites (Fig. 8F). Amino acids were also the most commonly observed metabolic group; however, in the high inoculum pressure treatment, amino acids were observed to be a smaller majority, leading to a more even metabolic fingerprint.

## Discussion

In this study, we observed reduced nematode egg counts with the addition of the amendments, collagen and chitin, for both soil types and for both levels of inoculum pressure. This highlights the potential of soil amendments to alter the soil microbiome and suppress *M. enterolobii* populations. An increase in *Meloidogyne* spp. inoculum is directly proportional to yield loss in a tomato plant [29]; thus, the ability to decrease inoculum load shows promise for future methods of biocontrol. Under standard inoculation conditions, collagen reduced nematode eggs per gram of root in agricultural soil, demonstrating its strong evidence for efficacy in nematode management, supporting results found by Galper et al. [10]. There was non-statistically significant evidence for the effect of chitin in agricultural soil (*P* = 0.67), but a consistent downward trend was noted, suggesting a potential suppressive effect that warrants further exploration. It is important to state that one possible explanation for such nematode reduction is due to an indirect effect on an increase in root biomass promoted by these amendments. Another possibility is due to the heavier weight of galls in the root tissue, which led to an increase in the root biomass, suggesting an actual reduction of nematodes per gram of infected root system. Moreover, we found that native and agricultural soils respond differently to the soil amendment. Both amendments exhibited strong evidence of effectiveness in native soil under standard inoculation. According to a study by Chialva et al. [30], the microbiome of native soils can induce a “state of alert” in tomatoes, enhancing responses to soilborne pathogens; however, further investigation would be necessary to conclude such an effect in our study.

Under high inoculum pressure, we observed a substantial decrease in eggs for both amendments in agricultural soil, affirming their aid in resistance against elevated disease challenges. It is important to note that high inoculum density can increase nematode competition, which may contribute to the observed decrease in eggs. Although reductions in native soil under these high-pressure conditions did not achieve statistical significance, the consistent trend of decreases across various conditions indicates a continued potential for nematode suppression. These findings indicate that the efficacy of collagen and chitin in reducing nematode populations may vary depending on soil type, and therefore different soil microbiomes, suggesting the indirect effect of the amendments in controlling the plant-parasitic nematode. Prior research has suggested that alterations in soil cultivation practices can impact soil suppressiveness and alter the microbial community to favor defense against *M. enterolobii* [4]. Thus, the soil microbes present in different soil types may differ in their use of collagen and chitin, impacting their efficacy against *M. enterolobii* infection.

Though we found evidence that suggests a microbial component in *M. enterolobii* control, it is important to also consider the role of chitin-induced resistance (CIR). Chitin, upon application, may have been recognized by the plant’s pattern recognition receptors (PRRs), which can initiate defense mechanisms against nematodes [31]. Interestingly, the systemic acquired resistance (SAR) pathway has been shown to be less effective against root-knot nematodes due to the nematode’s ability to suppress the expression of key PR genes [32]. However, due to the variability of CIR depending on host and pathogen species, different or additional defense pathways may be activated to provide a response against nematode infections [31]. The dual impact of beneficial microorganisms and CIR could synergistically contribute to the observed reduction in nematode egg counts. Further research into the CIR mechanism and its effectiveness against *M. enterolobii* in tomato could provide valuable insights into chitin not only enhancing microbial activity in the soil but also triggering directly plant immune responses, providing a comprehensive approach to nematode management.

There was strong evidence for increased plant biomass under both standard inoculation and high inoculum pressure conditions when soil was amended with collagen and chitin. These results suggest that the amendments alleviate plant stress through disease mitigation and potential biostimulatory effects. Chitin is known to enhance plant vigor and tolerance to stress, as documented by Pichyangkura and Chadchawan [33] and Shahrajabian et al. [34]. Similarly, collagen promotes plant growth when broken down into bioavailable forms by soil microorganisms, functioning as a biostimulant [35]. Increases in chlorophyll content noted in amended soils likely result from these biostimulatory effects. Liopa-Tsakalidi et al. [36] demonstrated that soil supplementation with chitin could elevate chlorophyll levels by up to 60%, which correlates with enhanced photosynthetic capacity and potentially improved plant yield. Likewise, collagen has been shown to boost chlorophyll production, thereby facilitating increased photosynthetic activity and overall plant health [35].

The additions of collagen and chitin, resulting in diminished microbial diversity and richness under both standard and high inoculum pressure conditions, signify a shift in the soil microbiome towards an increase in high-abundance taxa compared to a more balanced distribution (Fig. 7). The microbial community differentiation between amended and unamended groups is also highlighted in the principal coordinates analysis. This differentiation is primarily driven by shifts along principal coordinate 1, which captures the largest portion of the variance, indicating a strong influence on community composition driven by the amendments. The shift in microbial communities appears effective, particularly under high inoculum pressures, further suggesting that the amendments might be promoting microbial taxa that are suppressive to nematodes or otherwise beneficial for plant health.

In groups treated with chitin, a noticeable shift in the microbiome occurred, possibly due to the rise in chitin-degrading enzymes. In the standard inoculation group, there was an increase in *Streptomyces* when chitin was introduced into the soil. Moreover, under high inoculum pressure, this trend persisted, with *Streptomyces* experiencing even more substantial growth. Most streptomycetes are known to secrete chitinases [37], and many *Streptomyces* strains have been specifically identified for their production of chitin-degrading enzymes [38]. *Streptomyces* was the predominant genus in the native soil group; however, in agricultural soil, *Kitasatospora* dominated. Sawaguchi et al. [39] found that both *Streptomyces* and *Kitasatospora* exhibited strong increases with the addition of chitosan to the soil. Moreover, most chitosan-degrading bacteria isolated were identified as *Streptomyces*, strongly suggesting the involvement of these two groups in chitosan degradation. Specific strains of *Kitasatospora*, such as *Kitasatospora setae*, have been identified as producers of chitinases [39]. Hui et al. [40] identified *Bacillus* and *Kitasatospora* as opportunistic species in chitin degradation. *Bacillus* also increased in the collagen-amended soil, and many strains of *Bacillus* are known to produce collagenases [41]. Many collagenolytic *Bacillus* strains have been identified [42–44]. This increase in *Bacillus* when exposed to a collagen soil amendment may indicate an increase in collagenolytic *Bacillus* species. The increase of *Phialemonium* genera shown in the abundance and OTU-OTU networks (Fig. 3D and Fig. 6L, M, N, O, P) may be related to the ability of the *Phialemonium* to antagonize root-knot nematodes, as shown in previous research [45].

The network metrics in Table 5 indicate that the microbial communities in amended soils are influenced by these collagen and chitin treatments. Previous studies have highlighted the shift in microbial community dynamics when exposed to soil amendments [46–48]. In the study conducted by Zhou et al. [48], the group found that the addition of the biochar amendment increased microbial network complexity. In our study, under standard inoculation, the result mirrored findings by Zhou et al. [48], as collagen amendment in agricultural soil increased the number of both positive and negative edges. Conversely, the chitin amendment produced fewer overall connections, but the presence of negative edges in the OTU-OTU networks in the chitin-amended group may reflect selection for nematode-suppressive microbes. In native soils under the same conditions, collagen treatment results in a high number of positive edges and an absence of negative edges, which could be indicative of a network of mutualistic interactions. In native soils under the same conditions, collagen treatment results in a high number of positive edges and an absence of negative edges, indicative of a network of mutualistic interactions. Chitin introduces a notable number of negative edges, which may imply a shift towards a more competitive microbial environment, possibly favoring nematode antagonistic taxa. However, Poudel et al. [49] explain that the observed network structures in microbiome models can reflect a combination of biological interactions and shared environmental preferences, rather than direct mutualistic relationships. Thus, while the positive edges in collagen-treated soils suggest potential mutualistic interactions, they may also indicate taxa that thrive in similar environmental niches. In addition to showing a change in the soil microbial composition with the importation of soil amendments, we also saw a change in the soil microbial function with the metabolites assessed. Overall, these findings suggest strong evidence that the application of collagen and chitin amendments influences both the structure and function of soil microbial communities.

Under high inoculum pressure, the number of negative edges increased with collagen treatment in the agricultural soil, which could reflect an adaptive response of the microbial community under biotic stress, potentially directed towards inhibiting nematode infection. Chitin’s impact appears similar, with an increase in negative interactions that could influence microbial competition. In native soil experiencing high inoculum pressure, both collagen and chitin treatments have fewer total connections and a lower average degree, suggesting the establishment of a more specialized microbial network.

The bacterial phenotype-OTU network displays 36 OTUs (*P* < 0.05) associated with lower disease severity, highlighting the association between specific bacterial taxa and decreased disease incidence (Fig. 6A, D). Notably, two OTUs, *P. shaoguanensis* and *Streptomyces* sp., were both associated with lower disease incidence and identified as members of the core microbiome. This dual role underscores their potential importance in soil health and disease suppression. The identification of a larger number of significant bacterial OTUs compared to fungal OTUs can be attributed to several factors. Bacterial communities generally exhibit greater diversity and abundance, which might enhance the likelihood of detecting significant associations with disease resistance. Additionally, bacteria may respond more sensitively to inoculum pressure, or methodological biases might favor the detection and analysis of bacterial DNA over fungal DNA.

Conversely, the phenotype-OTU analysis for fungal communities revealed only 10 associations (*P* < 0.05) with low disease incidence (Fig. 7D). These fungal OTUs encompassed four different phyla, including three unidentified fungi. The phylum Mortierellomycotina was found in the highest abundance, whereas the strongest coefficient was attributed to Chytridiomycota. This lower number of significant fungal OTUs might be due to various factors such as lower diversity or methodological challenges in detecting and characterizing fungi in the microbiome.

The core microbiome consists of groups of microorganisms that establish essential interactions, which can be leveraged to enhance microbial functions [50]. The identification of core microbiomes in agricultural soils, comprising both bacterial and fungal constituents, offers promising avenues for enhancing plant health and disease management strategies, such as use in potential synthetic microbial communities [51]. The core microbes are favorable for such management strategies due to their strong interactions with plant functions and can aid in better recruitment and establishment of other beneficial microbes [50]. Among the 10,817 OTUs, this analysis identified 97 bacterial and 22 fungal OTUs as part of the “core microbiome” across different experimental conditions — under both standard inoculation and high inoculum pressure scenarios as depicted in Fig. 7C and F. The same analysis was conducted between soil types, revealing core bacterial (101 OTUs) and fungal (21 OTUs) communities across agricultural and native soil types (Fig. 7B, E). This conserved microbial cohort suggests a fundamental relationship between these taxa and plant health, irrespective of the level of disease pressure and soil type.

Untargeted soil metabolomic analysis has highlighted disparities between conditions of high inoculum pressure and standard inoculation. Under high inoculum pressure, there is a marked decrease in the expression of metabolic groups compared to standard inoculation conditions. This reduction may indicate a suppression or reallocation of metabolic functions, possibly as a response to stress or disease conditions in the soil environment [52]. The pronounced separation observed in the principal coordinates analysis (PCoA) underscores that the metabolic responses to high inoculum pressure differ significantly from those under standard conditions (Fig. 8B). This clustering could reflect adaptive or defensive modifications in the soil microbiome, which are vital for understanding soil resilience and health under stress [52].

Untargeted metabolomic analysis also gave substantial insights into the differential metabolic responses of native and agricultural soils under varying disease pressure. Amino acids and their metabolites were the predominant class identified in the metabolomic results, with this class being even more pronounced under standard inoculation conditions. An increase in amino acid concentration in the soil can be linked to soil microbial activity and nutrient uptake [53, 54]. In standard inoculation conditions, the native soil exhibited reduced metabolic activity. This difference may be due to the soil having a lower baseline metabolic activity due to inherent limitations in nutrient availability or microbial diversity due to less anthropogenic influence [4]. However, under high inoculum pressure, native soil demonstrated a notable increase in metabolic activity, suggesting a possible adaptive response to stress that aligns its metabolic profile more closely with that of agricultural soil.

This study represents the first comprehensive analysis of the response of the soil microbiome (bacteria and fungi) structure and function to chitin and collagen amendments under different soil conditions and inoculum pressure using *M. enterolobii* as a model soilborne parasite. The results of this study demonstrate the potential of collagen and chitin amendments for mitigating nematode infection and promoting overall plant health. Both amendments displayed efficacy in reducing nematode egg counts, with varying efficacy observed in each soil type, which highlights the importance of soil type in the microbiome response to amendments. The observed shifts in the soil microbiome suggest that these amendments may indirectly suppress nematodes by favoring the growth of specific microbial taxa. The identification of a core microbiome shared across treatments and soil types offers valuable insights for developing novel biocontrol agents against *M. enterolobii*. Future research should investigate the function of specific microbials in suppressing the nematode. Network analysis can help to identify potentially synergistic combinations of taxa [25, 49], since a potentially more effective strategy would be using a combination of isolates as a synthetic microbial community (SynComs) with complementary functions [3], considering both bacteria and fungi in SynComs has been shown to provide better results [55].

## Supplementary Information

Supplementary Material 1.

Supplementary Material 2.

Supplementary Material 3.

Supplementary Material 4.

## Data Availability

The raw read data used during the current study were submitted to NCBI SRA and are available under the accession number PRJNA1120455.

## Abbreviations

OTU: Operational Taxonomic Unit
PhONA: Phenotype-OTU network analysis
C: Control
N: Nematode
Co: Collagen
Ch: Chitin
NPK: Nitrogen-phosphorus-potassium
PPN: Plant-parasitic nematode
RFW: Root fresh weight
SDW: Shoot dry weight
rRNA: Ribosomal ribonucleic acid
ITS: Internal transcribed spacer
DNA: Deoxyribonucleic acid
ANOVA: Analysis of variance
PERMANOVA: Permutational multivariate ANOVA
PC1: Principal coordinate 1
PCoA: Principal coordinates analysis
CIR: Chitin-induced resistance
PRR: Pattern recognition receptors
SAR: Systemic acquired resistance
SynCom: Synthetic microbial community
