# Supplementary figures and images for: Underground guardians: how collagen and chitin amendments shape soil microbiome structure and function for Meloidogyne enterolobii control
Source: Microbiome. 2025 Jun 12;13:141. doi: 10.1186/s40168-025-02132-8 (PMC12160343; doi:10.1186/s40168-025-02132-8)

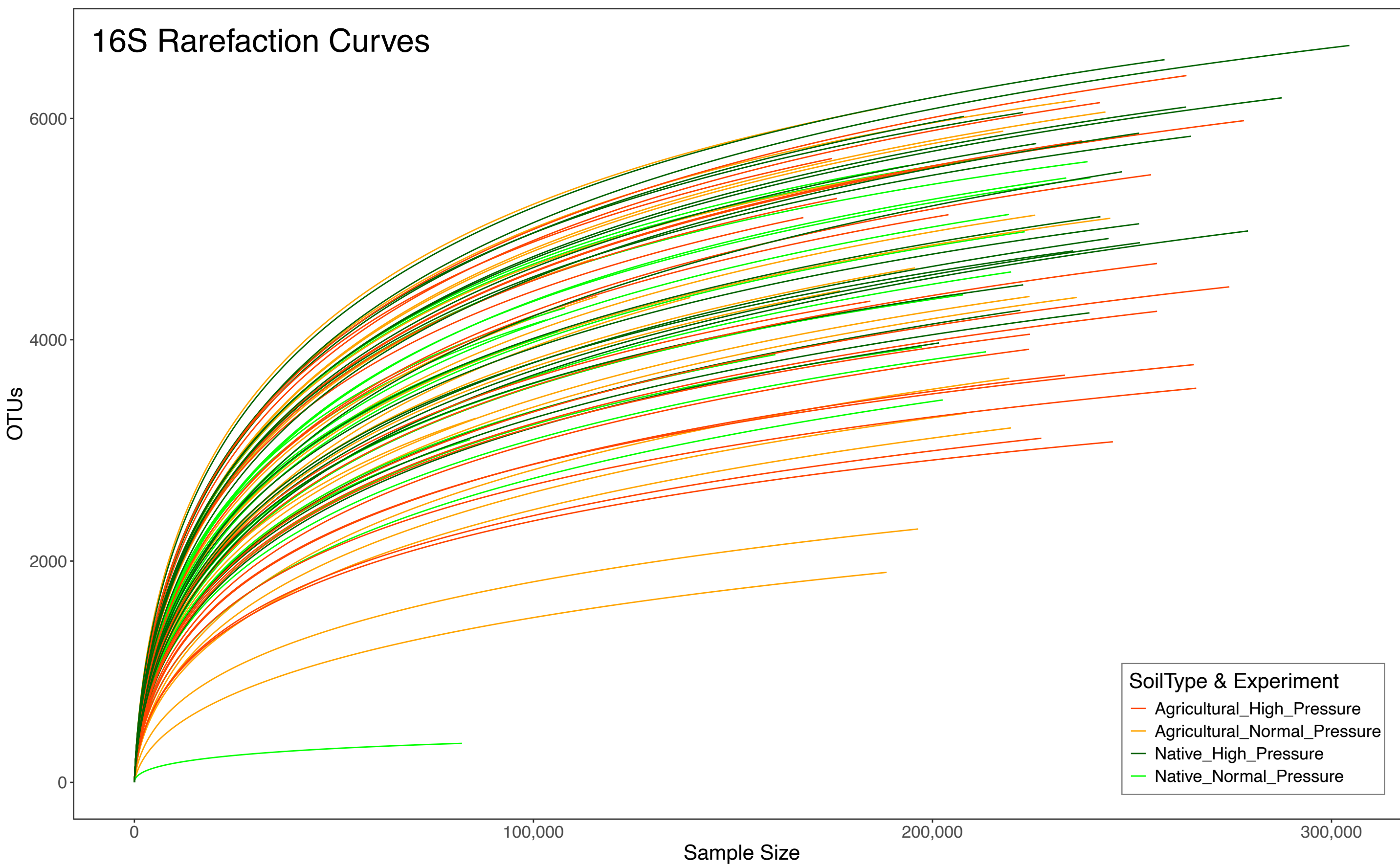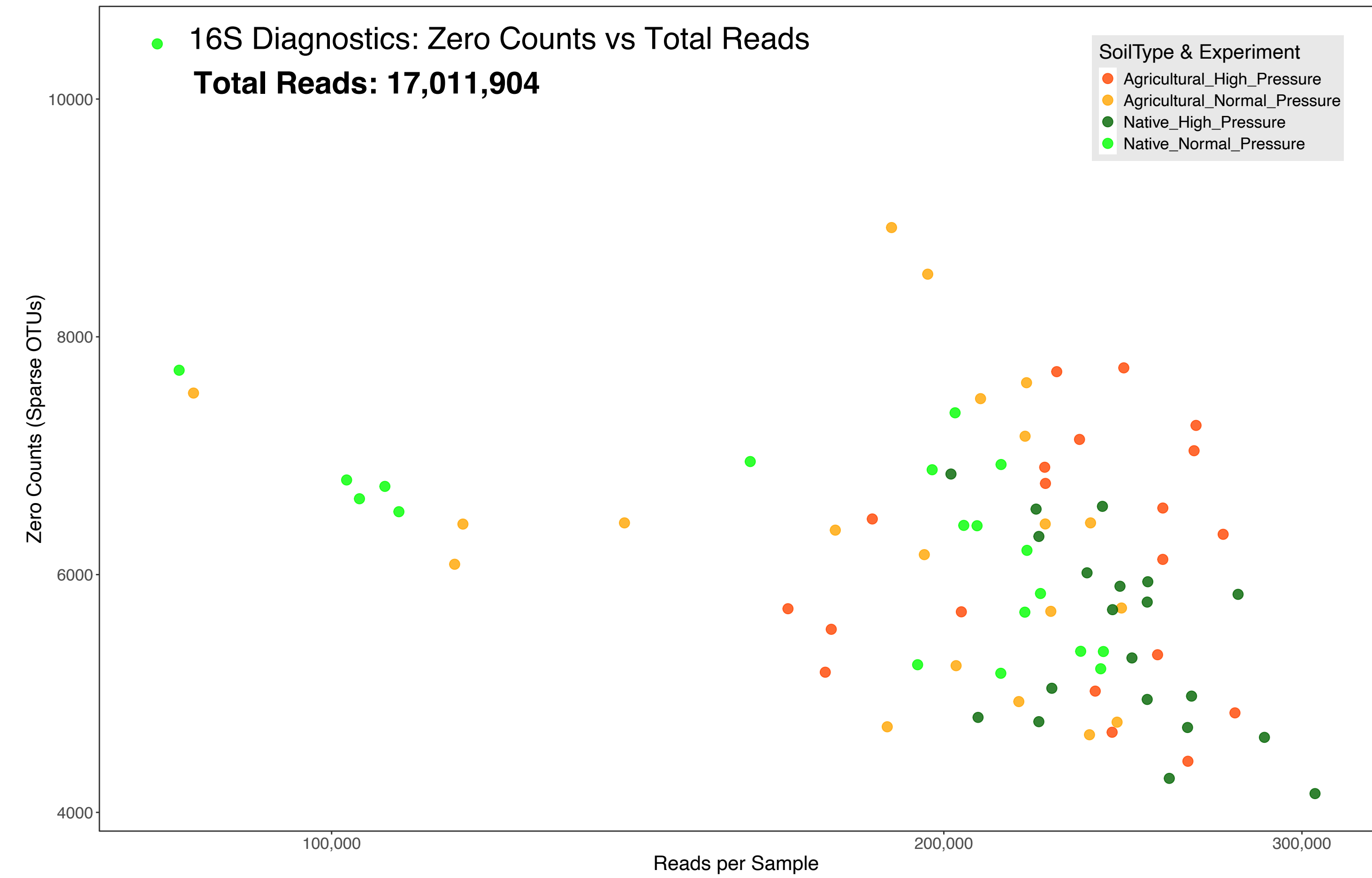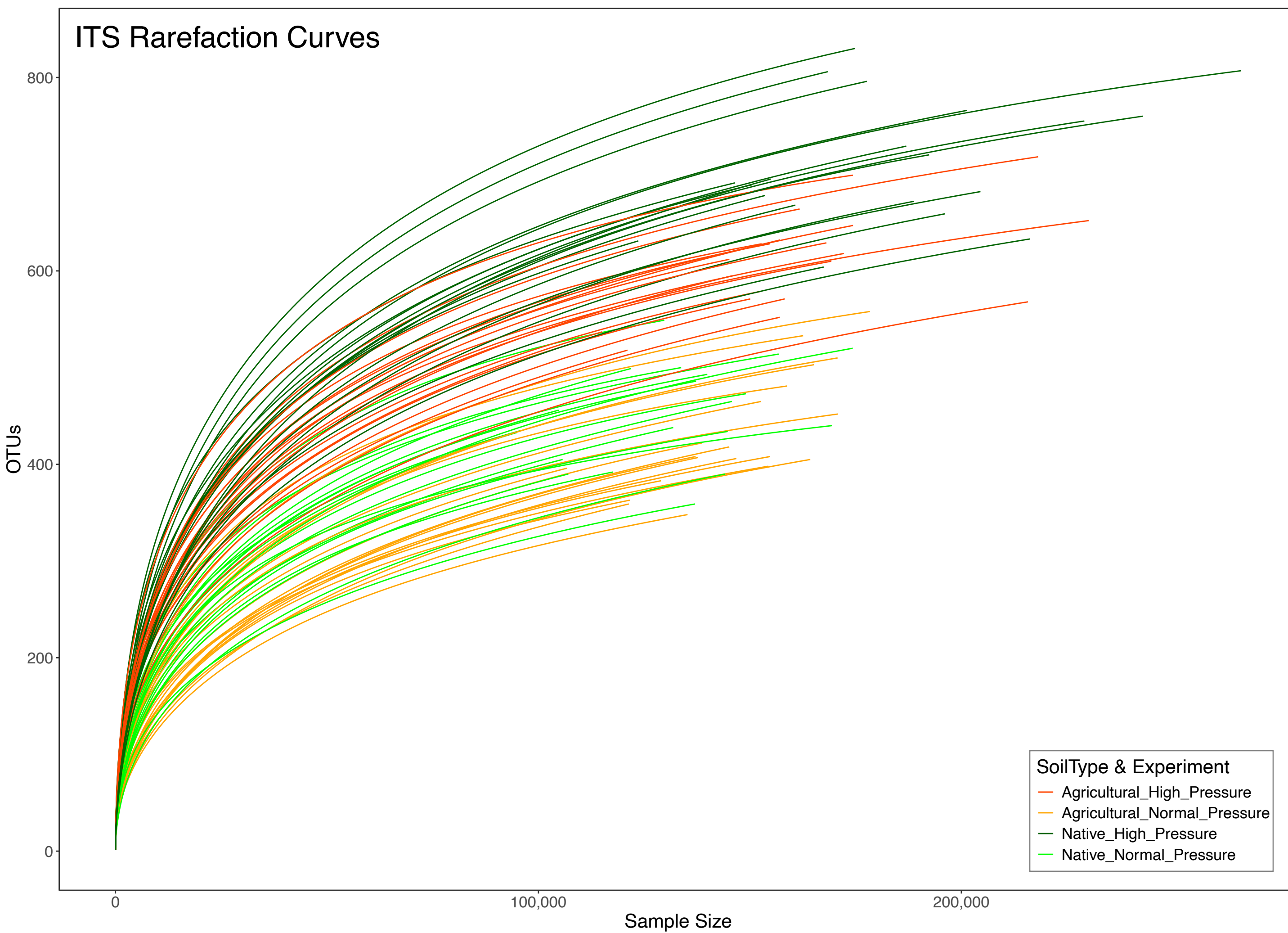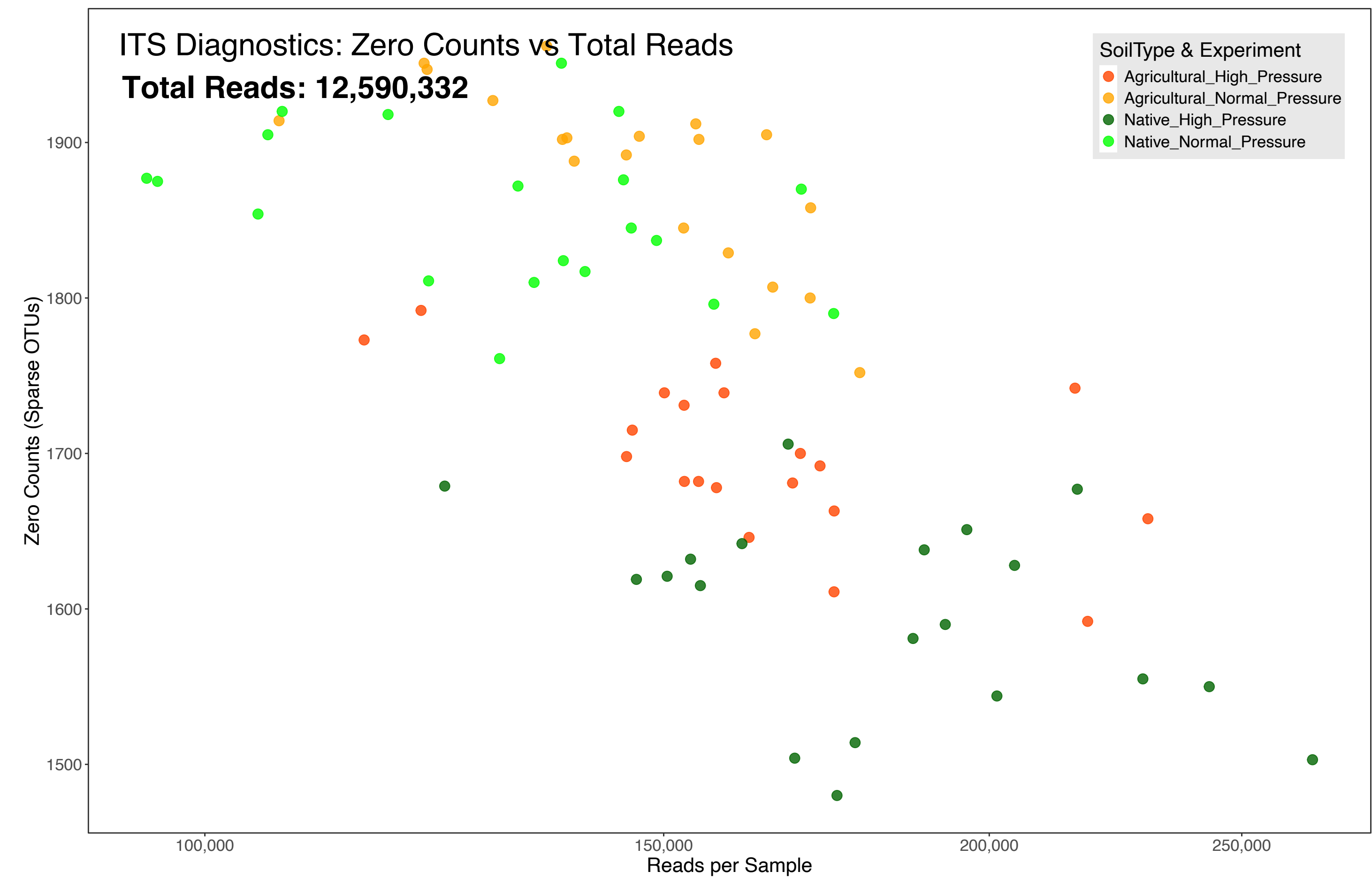

Supplement: Supplementary file 2 — Supplementary Material 1. [file 40168_2025_2132_MOESM1_ESM.pdf]
